# Supplementary material for: Deconstructing Immune Cell Infiltration in Human Colorectal Cancer: A Systematic Spatiotemporal Evaluation
Source: Genes (Basel). 2022 Mar 25;13(4):589. doi: 10.3390/genes13040589 (PMC9024576; doi:10.3390/genes13040589)
Supplement: Supplementary file 1 [file genes-13-00589-s001.zip › genes-1639763-SI.pdf]

Supplementary Table S1. Immune-based biomarker and immune checkpoint marker count data of colorectal cancer patients. Samples were measured in the following regions: main tumor mass (MAIN) and liver metastasis (MET). Unit of measure was the number of positive cells per 3.14 mm² (mean ± standard error).

	Parameter	CD3	CD4	CD8	CD20	CD23	CD45	CD56	CTLA-4	PD-L1	PD-1
	Type of sample										
	- MAIN	953.75 ± 136.50	12.40 ± 4.84	168.45 ± 25.46	168.42 ± 46.77	16.02 ± 5.00	811.83 ± 164.53	42.73 ± 7.06	136.18 ± 23.08	140.66 ± 33.18	57.49 ± 13.01
	- MET	846.43 ± 149.37	21.69 ± 5.35	193.61 ± 28.67	131.53 ± 49.31	13.86 ± 5.03	721.38 ± 173.57	18.08 ± 7.86	176.43 ± 27.87	65.74 ± 37.92	68.84 ± 14.91
MAIN	Sex										
	- Male	876.78 ± 177.40	13.59 ± 4.41	151.83 ± 26.56	83.71 ± 47.91	11.94 ± 7.85	790.06 ± 223.69	41.04 ± 12.62	118.47 ± 24.97	123.46 ± 52.96	48.28 ± 17.58
	- Female	890.15 ± 190.25	12.29 ± 4.65	184.79 ± 27.49	180.77 ± 46.73	23.82 ± 8.85	682.27 ± 246.15	44.11 ± 12.64	156.60 ± 26.54	169.60 ± 60.16	65.00 ± 20.56
	Sidedness										
	- Left-sided	798.22 ± 157.89	13.70 ± 3.90	168.29 ± 23.52	133.64 ± 41.25	20.19 ± 7.35	558.15 ± 203.24	41.59 ± 10.91	128.20 ± 22.15	109.67 ± 47.93	30.43 ± 15.97
	- Right-sided	1057.37 ± 224.60	11.46 ± 5.59	166.65 ± 33.05	133.05 ± 58.70	11.76 ± 9.84	1080.03 ± 276.42	44.59 ± 15.52	153.61 ± 32.16	215.02 ± 69.85	102.67 ± 22.03
	Lymph node status										
	- N0	760.61 ± 218.27	14.73 ± 5.49	197.57 ± 33.33	53.49 ± 56.20	10.33 ± 9.92	528.68 ± 287.86	59.21 ± 15.68	140.22 ± 30.79	133.42 ± 68.92	40.24 ± 23.36
	- N1	618.54 ± 216.79	14.16 ± 5.48	125.05 ± 32.09	81.30 ± 54.88	23.62 ± 10.62	500.71 ± 275.01	31.44 ± 14.89	145.27 ± 31.18	169.17 ± 66.96	83.61 ± 21.97
	- N1	1340.36 ± 230.84	9.83 ± 5.89	187.21 ± 34.24	288.47 ± 59.98	18.98 ± 10.47	1249.12 ± 292.47	38.71 ± 16.26	124.76 ± 34.32	128.54 ± 74.29	38.54 ± 24.52
	AJCC staging										
	- Stage I-II	689.79 ± 271.20	20.73 ± 6.72	191.83 ± 40.61	51.82 ± 70.21	11.31 ± 12.11	564.40 ± 347.46	71.61 ± 18.85	176.85 ± 37.54	188.14 ± 82.17	49.28 ± 27.99
	- Stage III	1122.86 ± 202.17	9.63 ± 5.12	145.15 ± 29.58	160.13 ± 53.07	25.99 ± 9.31	964.18 ± 256.89	37.96 ± 13.73	148.02 ± 29.40	174.49 ± 62.68	76.49 ± 20.51
	- Stage IV	746.33 ± 218.90	11.94 ± 5.25	181.85 ± 32.74	157.59 ± 56.66	11.39 ± 10.02	604.28 ± 283.78	30.00 ± 15.14	102.07 ± 29.74	81.46 ± 67.54	34.30 ± 23.03
MET	Sex										
	- Male	814.90 ± 195.52	18.44 ± 9.35	280.20 ± 48.00	93.73 ± 78.20	6.67 ± 2.11	634.91 ± 206.29	20.54 ± 6.04	192.65 ± 46.63	87.55 ± 37.90	68.07 ± 19.14
	- Female	715.37 ± 228.76	21.55 ± 10.98	177.52 ± 59.53	158.41 ± 90.92	5.95 ± 2.46	737.70 ± 251.52	16.16 ± 7.30	155.19 ± 52.28	28.19 ± 44.67	63.97 ± 23.08
	Sidedness										
	- Left-sided	819.26 ± 167.44	16.09 ± 7.91	196.14 ± 41.72	143.69 ± 66.48	6.40 ± 1.79	733.33 ± 177.03	16.74 ± 5.22	154.10 ± 38.42	68.26 ± 32.92	64.57 ± 16.32
	- Right-sided	603.04 ± 319.98	34.26 ± 15.74	196.13 ± 84.68	35.15 ± 130.14	6.23 ± 3.57	437.14 ± 361.96	26.35 ± 10.12	269.17 ± 79.40	43.11 ± 61.81	74.47 ± 34.22
	Lymph node status										
	- N0	897.37 ± 261.45	24.58 ± 12.40	198.68 ± 64.70	30.88 ± 106.03	4.27 ± 2.79	537.83 ± 286.36	29.01 ± 8.15	210.74 ± 60.63	43.09 ± 51.71	65.56 ± 27.65
	- N1	496.02 ± 245.66	10.00 ± 11.94	159.24 ± 61.87	111.80 ± 99.18	5.27 ± 2.61	561.16 ± 265.19	12.99 ± 7.73	198.11 ± 59.53	41.31 ± 48.37	65.69 ± 24.33
	- N1	906.28 ± 280.40	29.53 ± 14.13	217.42 ± 73.27	238.50 ± 114.51	11.37 ± 3.15	969.15 ± 312.51	13.60 ± 9.21	113.71 ± 68.74	113.80 ± 57.05	76.00 ± 28.25
	AJCC staging										
	- Stage I-II	1025.85 ± 377.91	42.24 ± 17.72	203.99 ± 90.12	27.05 ± 153.80	3.63 ± 4.15	718.97 ± 400.91	45.27 ± 11.38	226.18 ± 91.45	42.93 ± 73.18	73.73 ± 40.83
	- Stage III	895.88 ± 241.51	18.09 ± 11.87	234.46 ± 60.70	273.77 ± 99.59	8.62 ± 2.69	1053.91 ± 257.10	17.61 ± 7.61	187.56 ± 56.02	97.43 ± 46.28	73.19 ± 22.89
	- Stage IV	522.42 ± 225.34	13.98 ± 10.96	140.74 ± 58.06	33.18 ± 87.69	5.90 ± 2.43	296.59 ± 245.20	9.89 ± 6.87	153.38 ± 55.99	33.38 ± 46.96	62.05 ± 23.94

CTLA-4: cytotoxic T-lymphocyte-associated protein 4; PD-1: programmed cell death protein 1; PD-L1: programmed death-ligand 1.

Supplementary Table S2. Clinicopathological description of study participants, whose additional tumor regions were tested for immune markers.

Parameter	Number of observation / mean ± SD
Age (year)	61.92 ± 10.49
Sex (Male:Female)	29:23 (55.8%:44.2%)
Location of CRC [1]	
- Coecum	9 (17.3%)
- Ascending colon	11 (21.2%)
- Transverse colon	4 (7.7%)
- Descending colon	8 (15.4%)
- Sigmoid colon	12 (23.1%)
- Rectum	7 (13.5%)
Sidedness of the tumor	
- Right-sided	25 (48.1%)
- Left-sided	27 (51.9%)
pT – extent of the tumor	
- T1	0 (0%)
- T2	3 (5.8%)
- T3	38 (73.1%)
- T4	11 (21.2%)
pN – lymph node status	
- N0	17 (32.7%)
- N1	18 (34.6%)
- N2	17 (32.7%)
AJCC [9] staging	
- Stage I	0 (0%)
- Stage II	17 (32.7%)
- Stage III	24 (46.2%)
- Stage IV	11 (21.2%)

[1] No information about exact tumor location except for right-sided was available for one patient.
AJCC: American Joint Committee on Cancer; CRC: colorectal cancer.

[9] Jessup, J.; Goldberg, R.; Asare, E.; Benson, A.; Brierley, J.; Chang, G.; Chen, V.; Compton, C.; De Nardi, P.; Goodman, K., et al. Colon and Rectum. In *AJCC Cancer Staging Manual (8th Edition)*, Amin, M., Edge, S., Greene, F., Byrd, D., Brookland, R., Washington, M., Gershenwald, J., Compton, C., Hess, K., Sullivan, D., et al., Eds. Springer International Publishing: Chicago, IL, USA, 2018; pp. 251-274.

Supplementary Table S3. Immune-based biomarker and immune checkpoint marker count data in different areas of colorectal cancer and metastatic lymph node samples. Unit of measure was the number of positive cells per 3.14 mm^2 (mean ± standard error).

Parameter	MAIN	BORDER	FRONT	LN	NORMAL
CD3	1187.67 ± 488.82	1071.29 ± 666.15	676.99 ± 495.21	11381.40 ± 788.20 [1]	595.25 ± 598.68
CD4	7.74 ± 4.11	5.72 ± 5.11	3.89 ± 4.49	19.02 ± 6.55	14.80 ± 4.63
CD8	162.78 ± 74.37	184.39 ± 99.91	153.02 ± 79.78	1449.61 ± 114.85	137.56 ± 87.96
CD20	185.28 ± 412.79	187.00 ± 561.44	142.90 ± 446.30	10064.75 ± 691.45	198.12 ± 503.42
CD23	32.79 ± 26.74	8.06 ± 36.78	32.84 ± 28.19	420.63 ± 43.52	17.60 ± 33.38
CD45	952.74 ± 632.03	712.89 ± 833.49	439.09 ± 652.75	15606.50 ± 1108.65	522.10 ± 815.94
CD56	37.08 ± 11.63	50.64 ± 14.36	29.69 ± 12.38	17.32 ± 16.15	157.18 ± 12.87
CTLA-4	182.95 ± 37.43	167.35 ± 48.80	184.30 ± 39.15	445.67 ± 59.48	188.55 ± 47.29
PD-L1	237.04 ± 54.75	236.95 ± 70.37	103.59 ± 55.39	262.17 ± 88.94	71.32 ± 74.61
PD-1	84.06 ± 29.34	107.82 ± 40.48	88.04 ± 30.88	296.65 ± 39.99	87.55 ± 35.88

[1] $p < 0.0001$ vs. all groups.

MAIN: main tumor mass; BORDER: tumor-normal interface; FRONT: deepest infiltrative area; LN: lymph node metastasis; and NORMAL: normal colon tissue. CTLA-4: cytotoxic T-lymphocyte-associated protein 4; PD-1: programmed cell death protein 1; PD-L1: programmed death-ligand 1.

Supplementary Table S4. Clinicopathological description of study participants, whose sample was analyzed for the NanoString nCounter® PanCancer Immune Profiling Panel.

Parameter	Number of observation / mean ± SD
Age (year)	51.42 ± 11.61
Sex (Male:Female)	8:5 (61.5%:38.5%)
Location of CRC	
- Coecum	0 (0%)
- Ascending colon	2 (15.4%)
- Transverse colon	1 (7.7%)
- Descending colon	0 (0%)
- Sigmoid colon	5 (38.5%)
- Rectum	5 (38.5%)
Sidedness of the tumor	
- Right-sided	3 (23.1%)
- Left-sided	10 (76.9%)
pT– extent of the tumor	
- T1	0 (0%)
- T2	3 (23.1%)
- T3	9 (69.2%)
- T4	1 (7.7%)
pN – lymph node status	
- N0	3 (23.1%)
- N1	5 (38.5%)
- N2	5 (38.5%)
AJCC [9] staging	
- Stage I	0 (0%)
- Stage II	3 (23.1%)
- Stage III	7 (53.8%)
- Stage IV	3 (23.1%)

AJCC: American Joint Committee on Cancer; CRC: colorectal cancer.

[9] Jessup, J.; Goldberg, R.; Asare, E.; Benson, A.; Brierley, J.; Chang, G.; Chen, V.; Compton, C.; De Nardi, P.; Goodman, K., et al. Colon and Rectum. In *AJCC Cancer Staging Manual (8th Edition)*, Amin, M., Edge, S., Greene, F., Byrd, D., Brookland, R., Washington, M., Gershenwald, J., Compton, C., Hess, K., Sullivan, D., et al., Eds. Springer International Publishing: Chicago, IL, USA, 2018; pp. 251-274.

Supplementary Table S5. Details of significantly differentially expressed genes between main tumor mass and liver metastasis samples of colorectal cancer patients.

Gene abbreviation	Gene name	Gene Annotation	$\log_2$ fold change	Crude *p*-value
C4B	Complement C4B	Complement pathway<br>Innate immune response	–1.8028	0.0007
CARD9	Caspase recruitment domain family member 9	Innate immune response	0.6528	0.0007
CCR7	C-C motif chemokine receptor 7	CD molecules<br>Chemokines and receptors<br>Humoral immune response<br>Regulation of inflammatory response	1.4198	0.0008
CFI	Complement factor I	Innate immune response	–1.9313	0.0003
DEFB1	Defensin beta 1	Innate immune response	–1.6844	0.0006
IL1RAP	Interleukin-1 receptor accessory protein	Innate immune response	–1.1463	0.0005
IL27	Interleukin-27	Innate immune response<br>Interleukins	–1.6912	0.0002
LTB	Lymphotoxin beta	Cytokines and receptors<br>TNF superfamily members and their receptors	1.3115	0.0006
MBL2	Mannose binding lectin 2	Innate immune response	–2.5993	0.0001
MPPED1	Metallophosphoesterase domain containing 1	Basic cell functions	–2.1511	0.0008
TNFRSF8	Tumor necrosis factor receptor superfamily member 8	CD molecules<br>TNF superfamily members and their receptors	0.8854	0.0003

Supplementary Table S6. Details of differentially expressed genes of colorectal cancer patients, that were marginally different ($0.1 < p \leq 0.05$) after *p*-value adjustment.

Gene abbreviation	Gene name	Gene Annotation	$\log_2$ fold change	Crude *p*-value
ARG1 [1]	Arginase 1	Response to drug	–3.2496	0.0041
C3 [1]	Complement C3	Innate immune response<br>Regulation of immune response	–1.8868	0.0023
C5 [1]	Complement C5	Complement pathway<br>Innate immune response	–2.4838	0.0019
C8B [1]	Complement C8 Beta Chain	Complement pathway<br>Innate immune response	–2.8535	0.0042
CAMP [2]	Cathelicidin Antimicrobial Peptide	Inflammatory response	1.3052	0.0003
CCL16 [1]	C-C Motif Chemokine Ligand 16	Chemokines and receptors<br>Regulation of inflammatory response<br>Humoral immune response	–2.5082	0.0015
CCL24 [2]	C-C Motif Chemokine Ligand 24	Chemokines and receptors<br>Regulation of inflammatory response	–2.8245	0.0002
CCR5 [2]	C-C Motif Chemokine Receptor 5	CD molecules<br>Cytokines and receptors<br>T-cell polarization	0.8870	0.0007
CD19 [1]	CD19 Molecule	B-cell receptor signaling pathway<br>CD molecules<br>Regulation of immune response	1.0221	0.0052
CD22 [1]	CD22 Molecule	Adaptive immune response<br>CD molecules	1.2243	0.0052
CD36 [2]	CD36 Molecule	CD molecules<br>Receptors involved in phagocytosis	2.1267	0.0003
CD68 [2]	CD68 Molecule	Basic cell functions<br>CD molecules	1.2116	0.0004
CD79B [1]	CD79b Molecule	Adaptive immune response<br>B-cell activation<br>CD molecules	1.0022	0.0047
CFB [1]	Complement Factor B	Innate immune response	–1.9144	0.0050
CLU [1]	Clusterin	Innate immune response	–0.9334	0.0026
CR2 [1]	Complement C3d Receptor 2	B-cell activation<br>CD molecules<br>Innate immune response	1.0855	0.0026
CXCR5 [1]	C-X-C Motif Chemokine Receptor 5	Adaptive immune response<br>B-cell activation<br>CD molecules<br>Chemokines and receptors	0.9602	0.0051
DDX58 [1]	DExD/H-Box Helicase 58	Innate immune response	–0.4435	0.0043
F13A1 [1]	Coagulation Factor XIII A Chain	Basic cell functions	1.2811	0.0012

Supplementary Table S6. (cont.)

GZMB [1]	Granzyme B	Adaptive immune response<br>Basic cell functions<br>Cytotoxicity	1.2129	0.0031
HLA-DOB [1]	Major Histocompatibility Complex, Class II, DO Beta	Adaptive immune response<br>Antigen processing and presentation<br>Cytokines and receptors	1.1024	0.0022
HSD11B1 [1]	Hydroxysteroid 11-Beta Dehydrogenase 1	Basic cell functions	–2.2537	0.0010
MASP1 [1]	MBL Associated Serine Protease 1	Innate immune response	–1.8224	0.0030
SELL [1]	Selectin L	CD molecules<br>Regulation of immune response	1.1402	0.0028
SERPING1 [1]	Serpin Family G Member 1	Innate immune response	–1.5057	0.0023
SLAMF1 [1]	Signaling Lymphocytic Activation Molecule Family Member 1	CD molecules<br>Adaptive immune response	0.8093	0.0050
ST6GAL1 [1]	ST6 Beta-Galactoside Alpha-2,6-Sialyltransferase 1	Humoral immune response	–1.1028	0.0054
TNF [1]	Tumor Necrosis Factor	Humoral immune response<br>Interleukins<br>TNF superfamily members and their receptors	–0.6575	0.0057
TNFRSF13B [1]	Tumor Necrosis Factor Receptor Superfamily Member 13B	CD molecules<br>Chemokines and receptors<br>TNF superfamily members and their receptors	1.1543	0.0056
TNFRSF13C [1]	Tumor Necrosis Factor Receptor Superfamily Member 13C	CD molecules<br>Regulation of immune response<br>TNF superfamily members and their receptors	1.1401	0.0054
TNFRSF17 [1]	Tumor Necrosis Factor Receptor Superfamily Member 17	Basic cell functions<br>CD molecules<br>TNF superfamily members and their receptors	1.2917	0.0027
TNFSF10 [1]	Tumor Necrosis Factor Superfamily Member 10	CD molecules<br>Co-Regulators of autophagy and apoptosis/cell cycle<br>Cytokines and receptors<br>TNF superfamily members and their receptors	–0.6830	0.0029
TOLLIP [1]	Toll Interacting Protein	Innate immune response	–0.6791	0.0016
ZAP70 [1]	Zeta Chain Of T Cell Receptor Associated Protein Kinase 70	Adaptive immune response	1.0443	0.0014

[1] Differentially expressed between main tumor mass and liver metastasis samples. [2] Differentially expressed between right-sided and left-sided tumors.

Supplementary Table S7A. Results of the survival models on disease-specific survival. The 12 MAIN and 12 MET samples were analyzed separately using standard Cox regression models, while all of the 24 samples were analyzed in a mixed effect Cox regression model, where patient's IDs and sample source were used as the random and as the stratification factor, respectively.

DEG	Univariate models			Multivariate models		
	Hazard ratio	95% confidence interval	*p*-value	Hazard ratio	95% confidence interval	*p*-value
**MAIN samples**						
C4B	1.1230	0.6990 – 1.8030	0.6320	– [1]	– [1]	0.7600
CARD9	0.4485	0.1279 – 1.5730	0.2100	– [1]	– [1]	0.9150
CCR7	1.0200	0.5915 – 1.7590	0.9430	– [1]	– [1]	0.7850
CFI	1.0010	0.5369 – 1.8660	0.9980	– [1]	– [1]	0.8710
DEFB1	2.0930	0.7189 – 6.0920	0.1760	– [1]	– [1]	0.8610
IL1RAP	2.2640	0.5285 – 9.6980	0.2710	– [1]	– [1]	0.9100
IL27	1.5120	0.5634 – 4.0610	0.4120	– [1]	– [1]	0.8750
LTB	1.0890	0.6204 – 1.9120	0.7660	– [1]	– [1]	0.8730
MBL2	1.2500	0.7764 – 2.0140	0.3580	– [1]	– [1]	0.8580
MPPED1	1.1650	0.7211 – 1.8810	0.5330	– [1]	– [1]	0.6520
TNFRSF8	0.0247	0.0007 – 0.8121	**0.0378**	– [1]	– [1]	0.8840
**MET samples**						
C4B	0.6662	0.4269 – 1.0400	0.0737	– [1]	– [1]	0.8220
CARD9	2.9250	0.4420 – 19.3600	0.2660	– [1]	– [1]	0.9220
CCR7	1.3370	0.8015 – 2.2300	0.2660	– [1]	– [1]	0.8270
CFI	0.7571	0.5121 – 1.1190	0.1630	– [1]	– [1]	0.8290
DEFB1	0.8793	0.5222 – 1.4800	0.6280	– [1]	– [1]	0.9830
IL1RAP	0.6577	0.3318 – 1.3040	0.2300	– [1]	– [1]	0.9100
IL27	0.8067	0.5300 – 1.2280	0.3160	– [1]	– [1]	0.8970
LTB	1.3170	0.7409 – 2.3410	0.3480	– [1]	– [1]	0.9780
MBL2	0.7390	0.5311 – 1.0280	0.0727	– [1]	– [1]	0.9750
MPPED1	0.8168	0.5861 – 1.1380	0.2320	– [1]	– [1]	0.7470
TNFRSF8	7.2470	0.9618 – 54.6100	0.0546	– [1]	– [1]	0.9720
**Stratified, mixed effect models**						
C4B	0.9003	0.5010 – 1.6179	0.7300	0.2502	0.0754 – 0.8302	**0.0240**
CARD9	1.0342	0.1015 – 10.5376	0.9800	94.6416	2.3564 – 3801.1854	**0.0160**
CCR7	1.0835	0.3706 – 3.1677	0.8800	1.2032	0.2462 – 5.8804	0.8200
CFI	0.9128	0.5239 – 1.5906	0.7500	0.9184	0.2036 – 4.1426	0.9100
DEFB1	0.9353	0.3836 – 2.2803	0.8800	0.1164	0.0151 – 0.8971	**0.0390**
IL1RAP	0.8498	0.2315 – 3.1187	0.8100	2.0485	0.3548 – 11.8265	0.4200
IL27	0.8808	0.3168 – 2.4487	0.8100	5.3423	0.6231 – 45.8057	0.1300
LTB	1.0700	0.2723 – 4.1884	0.9200	0.3113	0.0434 – 2.2337	0.2500
MBL2	0.9410	0.6199 – 1.4285	0.7800	0.0626	0.0063 – 0.6229	**0.0180**
MPPED1	0.9378	0.5084 – 1.7300	0.8400	11.7669	0.9512 – 145.5605	0.0550
TNFRSF8	0.9864	0.0104 – 93.2655	1.0000	0.0008	0.0000 – 0.1723	**0.0093**

[1] Due to the low observation numbers, the model did not converge perfectly and the 95% confidence intervals were between 0 - ∞ for every DEGs.
DEG: differentially expressed gene; MAIN: main tumor mass; MET: liver metastasis.

Supplementary Table S7B. Results of the survival models on progression-free survival.

DEG	Univariate models			Multivariate models		
	Hazard ratio	95% confidence interval	Univariate *p*-value	Hazard ratio	95% confidence interval	Multivariate *p*-value
**MAIN samples**						
C4B	1.4470	0.8076 – 2.5940	0.2140	– [1]	– [1]	0.8620
CARD9	1.6180	0.4282 – 6.1140	0.4780	– [1]	– [1]	0.9230
CCR7	0.7471	0.4201 – 1.3290	0.3210	– [1]	– [1]	0.7460
CFI	1.2390	0.6233 – 2.4620	0.5410	– [1]	– [1]	0.9330
DEFB1	4.0390	1.0590 – 15.4100	**0.0410**	– [1]	– [1]	0.9030
IL1RAP	6.0500	0.6989 – 52.3700	0.1020	– [1]	– [1]	0.9630
IL27	0.7315	0.1800 – 2.9730	0.6620	– [1]	– [1]	0.8970
LTB	0.9176	0.5155 – 1.6330	0.7700	– [1]	– [1]	0.8590
MBL2	1.0410	0.5091 – 2.1300	0.9120	– [1]	– [1]	0.9250
MPPED1	1.2690	0.6265 – 2.5700	0.5080	– [1]	– [1]	0.8630
TNFRSF8	0.7123	0.2016 – 2.5160	0.5980	– [1]	– [1]	0.8190
**MET samples**						
C4B	0.6068	0.3703 – 0.9942	**0.0474**	– [1]	– [1]	0.8220
CARD9	40.4600	1.6900 – 968.9000	**0.0224**	– [1]	– [1]	0.9220
CCR7	1.6020	0.8164 – 3.1410	0.1710	– [1]	– [1]	0.8270
CFI	0.6606	0.4406 – 0.9906	**0.0449**	– [1]	– [1]	0.8290
DEFB1	0.8601	0.4694 – 1.5760	0.6260	– [1]	– [1]	0.9380
IL1RAP	0.3346	0.1271 – 0.8809	**0.0266**	– [1]	– [1]	0.9100
IL27	0.6606	0.4050 – 1.0770	0.0966	– [1]	– [1]	0.8970
LTB	2.0780	0.9875 – 4.3740	0.0540	– [1]	– [1]	0.9780
MBL2	0.7693	0.5807 – 1.0190	0.0677	– [1]	– [1]	0.9750
MPPED1	0.7612	0.5505 – 1.0520	0.0987	– [1]	– [1]	0.7470
TNFRSF8	2.3880	0.5146 – 11.0800	0.2660	– [1]	– [1]	0.9720
**Stratified, mixed effect models**						
C4B	0.7982	0.5087 – 1.2524	0.3300	0.5242	0.0478 – 5.7480	0.6000
CARD9	27.2715	3.4324 – 216.6779	**0.0018**	0.3186	0.0019 – 52.0571	0.6600
CCR7	0.9438	0.5316 – 1.6757	0.8400	0.0012	0.0000 – 1.0018	0.0500
CFI	0.6498	0.3844 – 1.0984	0.1100	11.1189	0.8072 – 153.1549	0.0720
DEFB1	1.2673	0.6808 – 2.3591	0.4600	26.5890	1.1223 – 629.9572	**0.0420**
IL1RAP	0.3519	0.1179 – 1.0501	0.0610	5.2422	0.0784 – 350.5389	0.4400
IL27	0.5981	0.3165 – 1.1301	0.1100	0.1069	0.0157 – 0.7283	**0.0220**
LTB	1.2004	0.6760 – 2.1314	0.5300	640.2143	1.5515 – 264171.4945	**0.0350**
MBL2	0.7291	0.5054 – 1.0518	0.0910	0.5086	0.0489 – 5.2926	0.5700
MPPED1	0.7463	0.4892 – 1.1383	0.1700	0.0304	0.0004 – 2.1048	0.1100
TNFRSF8	1.5815	0.4790 – 5.2221	0.4500	0.3266	0.0014 – 78.2373	0.6900

[1] Due to the low observation numbers, the model did not converge perfectly and the 95% confidence intervals were between 0 - ∞ for every DEGs.
DEG: differentially expressed gene; MAIN: main tumor mass; MET: liver metastasis.

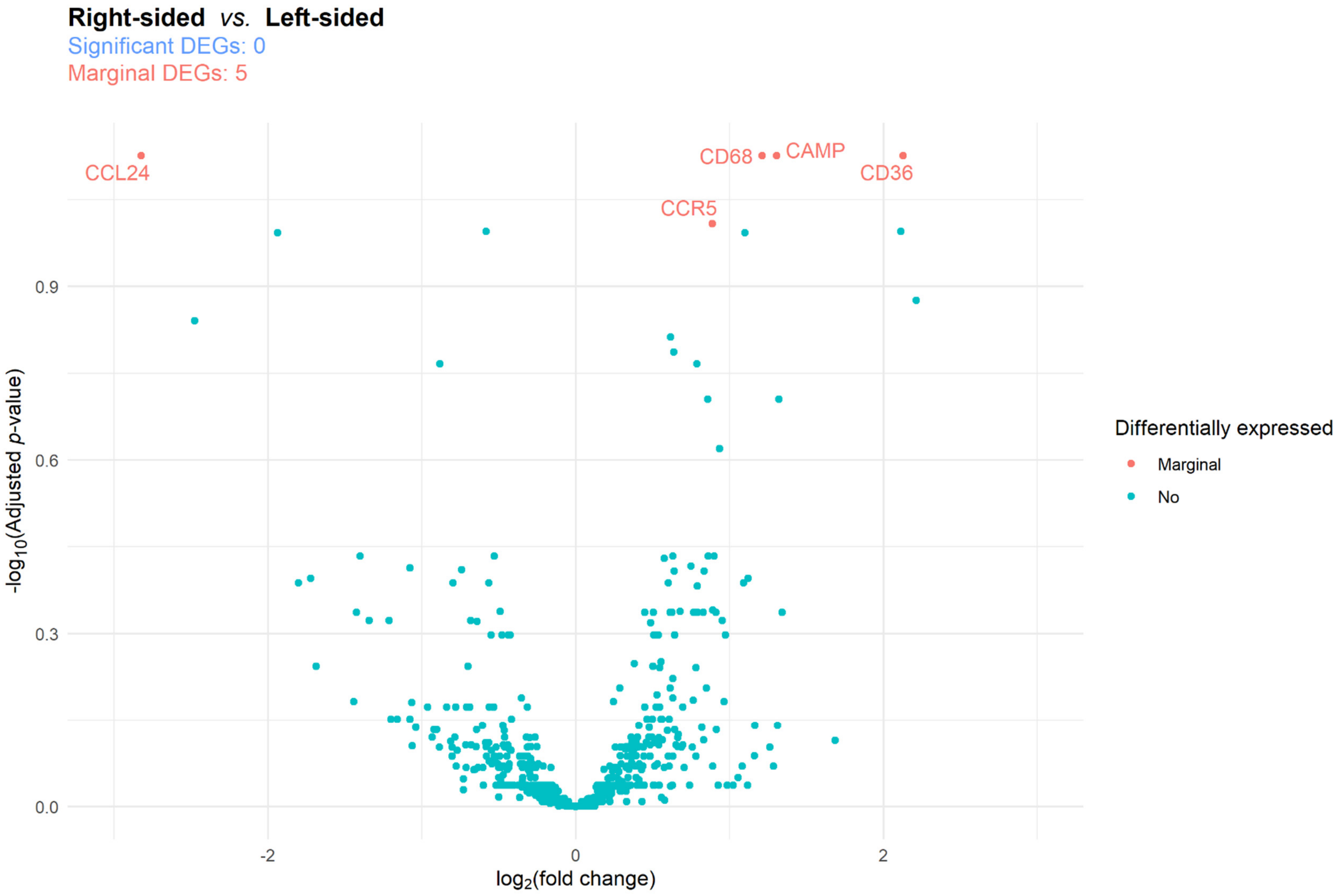

Figure S1. Differentially expressed genes (DEGs) between right-sided and left-sided tumor samples of colorectal cancer patients. The false discovery rate method was used for *p*-value adjustment.
